# The effect of “Nutramil^TM^ Complex,” food for special medical purpose, on breast and prostate carcinoma cells

**DOI:** 10.1371/journal.pone.0192860

**Published:** 2018-02-14

**Authors:** Aneta A. Koronowicz, Mariola Drozdowska, Barbara Wielgos, Ewelina Piasna-Słupecka, Dominik Domagała, Joanna Dulińska-Litewka, Teresa Leszczyńska

**Affiliations:** 1 Department of Human Nutrition, Faculty of Food Technology, University of Agriculture, Krakow, Poland; 2 Olimp Laboratories sp. z o.o., Dębica, Poland; 3 Chair of Medical Biochemistry, Jagiellonian University Medical College, Kraków, Poland; University of South Alabama Mitchell Cancer Institute, UNITED STATES

## Abstract

Nutramil^TM^ Complex is a multicomponent food product that meets the requirements of a food for special medical purpose. As a complete, high-energy diet it consists of properly balanced nutrients, vitamins and minerals. The aim of this study was to assess the effect of Nutramil^TM^ Complex on breast and prostate carcinoma cells. Our results showed that Nutramil^TM^ Complex reduced the viability and proliferation of breast and prostate cancer cells and that this process was associated with the induction of apoptosis via activation of caspase signalling. Data showed elevated levels of p53 tumour suppressor, up-regulation of p38 MAPK and SAPK / JNK proteins and downregulation of anti-apoptotic ERK1/2, AKT1 and HSP27. Treatment with Nutramil^TM^ Complex also affected the expression of the BCL2 family genes. Results also showed down-regulation of anti-apoptotic *BCL-2* and up-regulation of pro-apoptotic members such as *BAX*, *BAD*, *BID*. In addition, we also observed regulation of many other genes, including Iκβα, Chk1 and Chk2, associated with apoptotic events. Taken together, our results suggest activation of the mitochondrial apoptotic pathway as most likely mechanism of anti-carcinogenic activity of Nutramil^TM^ Complex.

## Introduction

Breast cancer is one of the most common type of cancer affecting women around the world [1], and is also the leading cause of cancer death in the female part of the population [2]. Prostate cancer, on the other hand, occupies the third place of type of cancer for males, and fourth place as the cause of cancer deaths [3]. Although improving diagnostic tools helps with the patients’ outcome, research in cancer prevention remains insufficient.

Numerous studies showed that selected nutrients and non-nutrients can act preventively in cancer. They included, among others, diets rich in vegetables (i.e. cruciferous vegetables, tomatoes), fruits, oily sea fish and soybeans; thus, rich in vitamin E, C, carotenoids (beta-carotene, lycopene) selenium, omega-3 fatty acids, glucosinolates, polyphenols and etc. [4–6].

Nutramil^TM^ Complex is a Food for Special Medical Purpose (FSMP), intended for people whose nutritional requirements cannot be met by normal food, which follows the European Union Directive 1999/21/EC regarding its composition and levels of vitamins and minerals. It can be used as a complete, all-day meal for the hospitalised patient, or used as a supplement. The choice depends on nutritional status, individual energy needs, ability to take other meals as well as general health or disease progression. Nutramil^TM^ Complex is also used to prevent malnutrition in associated with lack of appetite, anorexia, Alzheimer's disease, Parkinson's disease, cancer, irritable bowel syndrome, partial gastric or intestinal resection, etc. In addition, it is also gluten- and lactose-free.

In our pilot studies, Nutramil^TM^ Complex has decreased the viability of breast and prostate cancer cells [7, 8]. To our knowledge, there are no other reports showing Nutramil^TM^ anti-cancer properties as a multi-component product. However, limited data is available on some individual substances that constitutes the composition of Nutramil^TM^ Complex. In this manuscript, we present the effect of Nutramil^TM^ Complex on breast and prostate carcinoma cells. Our results suggest that observed reduction in viability of cancer cells can be associated with induction of apoptosis.

## Materials and methods

### Testing material

Composition of the Nutramil^TM^ Complex (NC) is given in the S1 Table. To determine the effect of complete compound on cancer cells, two additional compounds were investigated: an incomplete Nutramil^TM^ Complex without calcium caseinate (NC-CC) and calcium caseinate alone (CC) as one of the main components of the formulation. Analyses were blinded as all samples were numerically encoded. Their decoding took place at the end of the study.

### Cell culture

The human breast adenocarcinoma cell line MCF-7 (estrogen receptor (ER) positive cell line, progesterone receptor positive and HER2 negative, ATCC® HTB­22^TM^) was purchased from the American Type Culture Collections. Cells were cultured in appropriate EMEM medium (Sigma-Aldrich, MO, USA) according to the ATCC protocol with an addition of 10% FBS (Sigma-Aldrich, MO, USA).

The human prostate carcinoma DU145 (not detectably hormone sensitive, ATCC® HTB-81^TM^) and LNCaP cell lines (androgen receptor, positive; estrogen receptor, positive; ATCC® CRL-1740™) were purchased from the American Type Culture Collections. Cells were cultured in appropriate EMEM and RPMI 1640, respectively, medium (Sigma-Aldrich, MO, USA) according to the ATCC protocol with an addition of 10% FBS (Sigma-Aldrich, MO, USA).

The human normal prostate PNT-2 cell line was purchased from HPA Culture Collections (Sigma-Aldrich, MO, USA). Cells were cultured in appropriate RPMI 1640 medium (Sigma-Aldrich, MO, USA) according to the protocol with an addition of 10% FBS (Sigma-Aldrich, MO, USA).

### Cell treatment

Cells were seeded on 96-well plates (8x10^3^ cells per well), 12-well plates (9x10^4^ cells per well) or 6-well plates (2x10^5^ cells per well). 24 h after seeding, growth medium was replaced with a medium containing: 1) NC, 2) NC-CC or 3) CC. The final applied concentrations of each treatment were 1, 2, 3, 4, 5 and 10% for 24, 48 and 72 h. Untreated cells in culture medium were used as a negative, untreated control (UC).

For apoptosis assessment, Staurosporine (Sigma-Aldrich, MO, USA) was used as a positive control at the final concentration of 1.5 μM.

### Cytotoxicity and cell viability

Cell viability was determined by Crystal Violet Assay (Sigma-Aldrich, MO, USA) and Cytotoxicity Detection Kit LDH (Roche, Poland) according to the manufacturer's protocol.

Each treatment included 3 biological and 4–5 technical replicates.

### Cell proliferation assessment

Cell proliferation was determined with 5’-bromo-2’-deoxy-uridine (BrdU) Labeling and Detection Kit III (Roche), according to manufacturer's instruction.

Each treatment included 3 biological and 3 technical replicates.

### RT and Real-time PCR analysis

Reverse transcription was performed using 1 μg of total RNA isolated from the cells using the Maxima first Strand cDNA Synthesis kit for RT-qPCR (Thermo Scientific). A quantitative verification of genes was performed with TaqMan® Array Human C-MYC and Apoptosis (Thermo Fisher Scientific) according to the manufaturer’s protocol and StepOnePlus™ system. Analysed genes: *AKT1*, *APAF1*, *BAD*, *BAX*, *BCL2*, *BID*, *CASP3*, *CASP8*, *CDKN2A*, *CYCS*, *FADD*, *FAS*, *FASLG*, *HRAS*, *IGF1*, *IGF1R*, *KRAS*, *MYC*, *NRAS*, *RRAS*, *TP53*, *YWHAB*, *YWHAE*, *YWHAG*, *YWHAH*, *YWHAQ*, *YWHAZ*. Results were normalized using at least two reference genes (*18S*, *GAPDH*, *HPRT1or GUSB)* and were calculated using the 2^-ΔΔC^T method [9].

### Stress and apoptosis signalling assay

Cell extracts were prepared and analyzed using the PathScan® Stress and Apoptosis Signaling Antibody Array Kit (Chemiluminescent Readout) #12856, Cell Signaling Technology, MA, USA. Assay target proteins were P44/42 MAPK (ERK1/2) phosphorylation, AKT phosphorylation, BAD phosphorylation, HSP27 phosphorylation, SMAD2 phosphorylation, p53 phosphorylation, p38 MAPK phosphorylation, SAPK/JNK phosphorylation, PARP cleavage, Caspase-3 cleavage, Caspase-7 cleavage, total Ikβα, Chk1 Ser345 phosphorylation, Chk2 phosphorylation, Ikβ α phosphorylation, eIF2α phosphorylation, TAK1 phosphorylation, Survivin and α-Tubulin as a reference protein.

Images were acquired by briefly exposing the slide to standard chemiluminescent film. Densitometry analysis was performer using ImageJ (http://imagej.nih.gov/ij/). Results are shown as a mean±SD normalized to the internal reference protein (α-Tubulin). Untreated negative control (UC) was set as 100% expression level.

### Western blot assay

Whole cell lysis was carried out using Cell Lysis Buffer (Cell Signaling Technology, MA, USA) according to the manufacturer’s protocol, with the addition of Protease Inhibitor Cocktail (BioShop, Canada). Total protein was quantified with Pierce BCA^TM^ Protein Assay Kit (Thermo Fisher Scientific, MA, USA). Protein extract was separated on a polyacrylamide gel and transferred to a nitrocellulose filter (Bio-Rad, CA, USA) by wet-electroblotting. Subsequently, the immobilized proteins were incubated with the appropriate primary antibody: cytochrome c (#11940), Smac/Diablo (#2954), HtrA2/Omi (#9745) and β-Tubulin (#2128) (Cell Signaling Technology, MA, USA). Finally, the appropriate secondary antibody conjugated with horseradish peroxidase (#7074, Cell Signaling Technology, MA, USA) was applied. Detection was executed by chemiluminescence, using Clarity™ Western ECL Substrate (Bio-Rad, CA, USA). Western blot stripping buffer (Thermo Scientific, MA, USA) was used to remove the antibodies from the membrane.

### Statistical analysis

All experiments were performed in at least three independent experiments and measured in triplicates. Shapiro-Wilk’s test was applied to assess normality of distribution. An independent samples t-test was applied to compare unpaired means between two groups and P≤0.05 was considered statistically significant. All analyses were performed using Statistica ver.12 (StatSoft, Tulsa, OK, USA).

## Results

### Cytotoxicity

Nutramil^TM^ Complex showed a cytotoxic effect on all examined cells, both cancer and non-malignant in a dose–and time–dependent manner (Table 1). Cytotoxicity levels for NC-CC were lower than those observed for NC (Table 1). All cell lines showed mostly necrotic changes at 10% concentration of NC (Table 1). The cytotoxicity results for treatment with 5% NC was at approximately 15% of UC after 24 hrs for all cell lines. Based on those results, all further experiments were performed using 4% concentration for NC as well as NC-CC, that lowered proliferation but did not caused a significant necrosis to cells.

**Table 1:** Cytotoxicity of Nutramil^TM^ Complex.

Concentration	DU145 Cytotoxicity %		LNCaP Cytotoxicity %		MCF-7 Cytotoxicity %		PNT-2 Cytotoxicity %	
	NC vs UC ± SD	NC-CC vs UC ± SD	NC vs UC ± SD	NC-CC vs UC ± SD	NC vs UC ± SD	NC-CC vs UC ± SD	NC vs UC ± SD	NC-CC vs UC ± SD
**24 h**								
**1%**	0,04 ± 0,20	Nt	2,88 ± 0,51	Nt	4,69 ± 1,21	Nt	4,26 ± 0,39	Nt
**2%**	2,08 ± 0,80	Nt	5,46 ± 1,61	3,10 ± 1,33	5,86 ± 0,81	Nt	5,98 ± 0,39	Nt
**3%**	6,18 ± 0,40	3,10 ± 0,33	8,55 ± 1,11	1,15 ± 0,84	8,41 ± 0,83	9,15 ± 0,88	10,75 ± 4,10	1,67 ± 0,90
**4%**	9,67 ± 1,47	1,15 ± 0,84	11,24 ± 0,83	4,20 ± 1,22	12,41 ± 0,67	9,83 ± 0,92	13,30 ± 2,27	1,06 ± 0,14
**5%**	15,21 ± 1,33	4,20 ± 1,22	15,47 ± 2,33	Nt	18,04 ± 2,27	12,99 ± 0,73	15,16 ± 2,54	1,48 ± 0,52
**10%**	28,34 ± 2,06	Nt	30,22 ± 6,25	Nt	20,70 ± 1,24	Nt	29,11 ± 7,50	Nt
**48 h**								
**1%**	0,70 ± 0,10	Nt	2,76 ± 2,74	Nt	3,61 ± 2,06	Nt	2,45 ± 2,99	Nt
**2%**	1,69 ± 0,36	Nt	5,97 ± 7,14	Nt	4,07 ± 0,80	Nt	2,31 ± 2,06	Nt
**3%**	7,52 ± 1,57	6,81 ± 1,73	7,70 ± 3,19	6,62 ± 0,34	6,36 ± 1,66	7,48 ± 1,60	9,84 ± 3,80	2,48 ± 0,23
**4%**	10,24 ± 1,89	7,88 ± 0,46	8,06 ± 1,25	6,38 ± 0,75	9,12 ± 3,03	8,72 ± 2,50	19,68 ± 12,29	3,71 ± 0,16
**5%**	13,83 ± 1,78	11,60 ± 2,24	11,30 ± 1,92	4,53 ± 0,40	10,00 ± 0,52	11,35 ± 1,25	22,64 ± 11,33	2,04 ± 0,95
**10%**	36,97 ± 4,32	Nt	22,68 ± 3,75	Nt	18,22 ± 7,37	Nt	44,23 ± 12,89	Nt
**72 h**								
**1%**	0,58 ± 0,04	Nt	0,81 ± 2,39	Nt	3,93 ± 2,45	Nt	1,79 ± 0,77	Nt
**2%**	0,12 ± 0,83	Nt	1,80 ± 2,38	Nt	5,03 ± 3,14	Nt	1,13 ± 0,35	Nt
**3%**	4,00 ± 0,19	1,28 ± 0,86	7,18 ± 2,40	7,29 ± 0,55	7,03 ± 4,26	5,61 ± 1,18	5,46 ± 0,88	3,81 ± 0,33
**4%**	8,08 ± 1,24	2,85 ± 0,18	8,90 ± 5,29	4,88 ± 0,67	7,16 ± 3,50	5,14 ± 1,23	5,01 ± 1,13	4,25 ± 1,17
**5%**	18,78 ± 2,16	6,72 ± 1,59	11,55 ± 6,95	4,28 ± 0,28	9,59 ± 6,34	7,03 ± 1,49	6,56 ± 1,37	2,98 ± 1,45
**10%**	42,75 ± 4,16	Nt	27,74 ± 8,53	Nt	27,92 ± 6,99	Nt	21,07 ± 5,12	Nt

MCF-7 breast cancer cells; DU145 and LNCaP prostate cancer cells; PNT-2 normal cancer cells were seeded on the 96-well plates (8x10^3^ cells per well). 24 h after, growth medium was replaced with a medium containing “Nutramil^TM^ Complex” and “Nutramil^TM^ Complex” without calcium caseinate (1–10% of concentration, 24–72 h). Cytotoxicity was measured with Cytotoxicity Detection Kit LDH (Roche, Poland). Values are expressed as mean ± SD for n = 15, standardized to untreated control (UC) as 100%. Nt, no treatment.

### Cell viability

**Fig 1:**
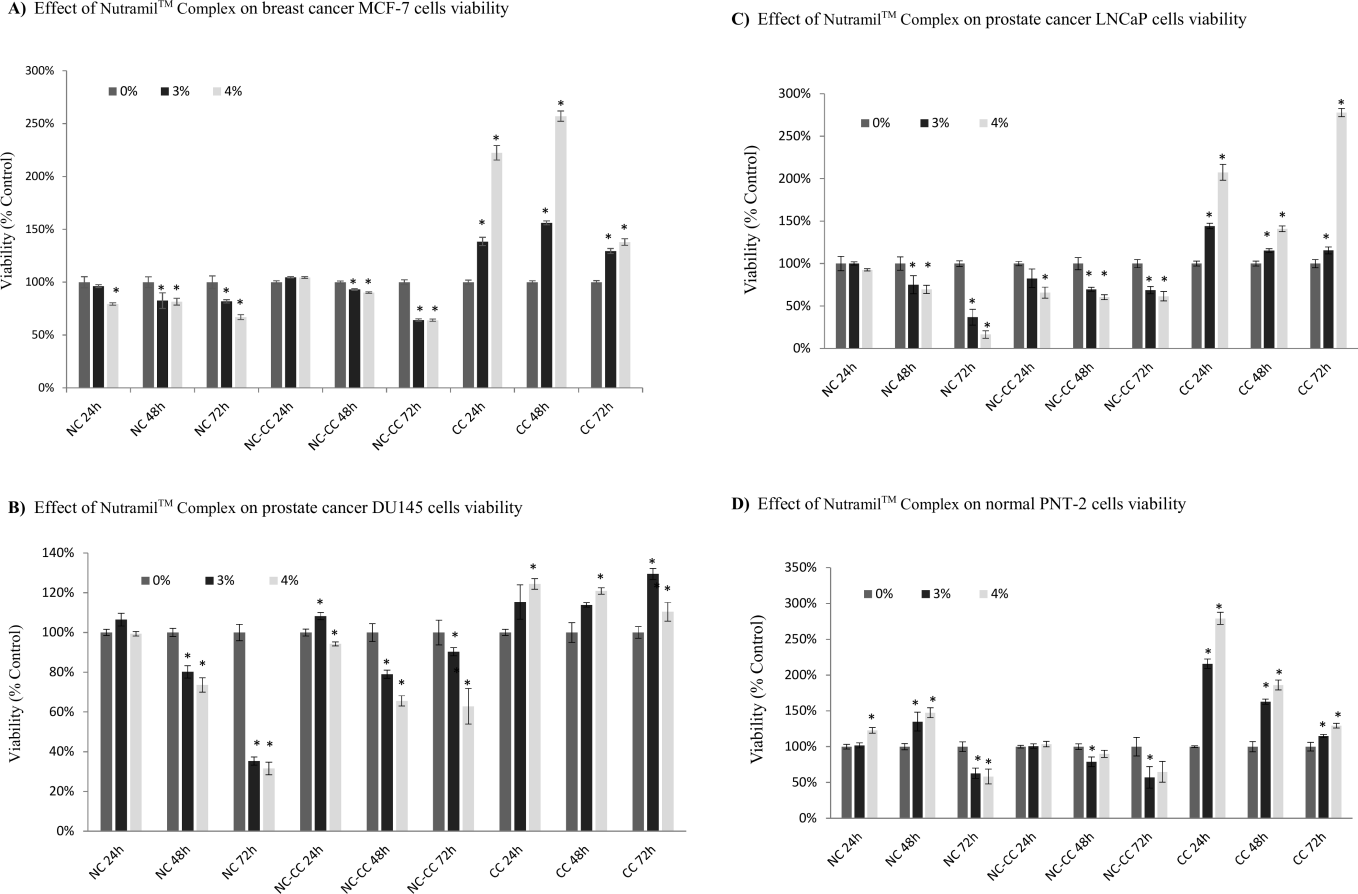
Effect of Nutramil^TM^ Complex on cells viability. Cells of MCF-7 breast cancer line (A); DU145 prostate cancer line (B); LNCaP prostate cancer line (C) and PNT-2 normal prostate line (D) were seeded on the 96-well plates (8x10^3^ cells per well). 24 h after, growth medium was replaced with a medium containing Nutramil^TM^ Complex (NC) or Nutramil^TM^ Complex without calcium caseinate (NC-CC) or calcium caseinate (CC) at concentration 0, 3, 4% for 24–72 h. Cell viability was measured with Cristal Violet (Sigma-Aldrich, Poland). Values are expressed as mean ± SD for n = 12, standardized to untreated control (UC) as 100%. Statistical significance was based on t-test *P≤0.01 vs. UC.

Treatment with 4% Nutramil^TM^ Complex decreased the viability of MCF-7 breast cancer cell line by 20% after 24–48 h and by 35% after 72 h (P≤0,001; Fig 1A). Results for NC-CC showed a similar trend, while CC showed quite the opposite—significant increase in the MCF-7 cells viability (P≤0.001; Fig 1A).

Treatment with 4% Nutramil^TM^ Complex also decreased the viability of prostate cancer cells by 30% after 48 h and by 70% after 72 h (P<0,001, Fig 1B and 1C). The results did not differ significantly between androgen-independent DU145 cell line and androgen-dependent LNCaP. On the other hand, NC treatment increased significantly the viability of the normal prostate cells PNT-2 after 24–48 h to 150% of the UC. Interestingly, after 72 h viability sharply decreased to 60% of UC (P≤0.001; Fig 1D).

DU145 and LNCaP cells treated with 4% NC-CC showed a significant, 30–40% reduction in viability (P≤0.001; Fig 1B and 1C). Viability of PNT-2 cells was not affected by NC-CC after 24 and 48 h, and decreased after 72 h by 30–35% (P≤0.001; Fig 1D). Treatment with calcium caseinate cells showed a significant increase in cell viability across all tested cell lines (Fig 1A–1D).

### Cell proliferation

**Fig 2:**
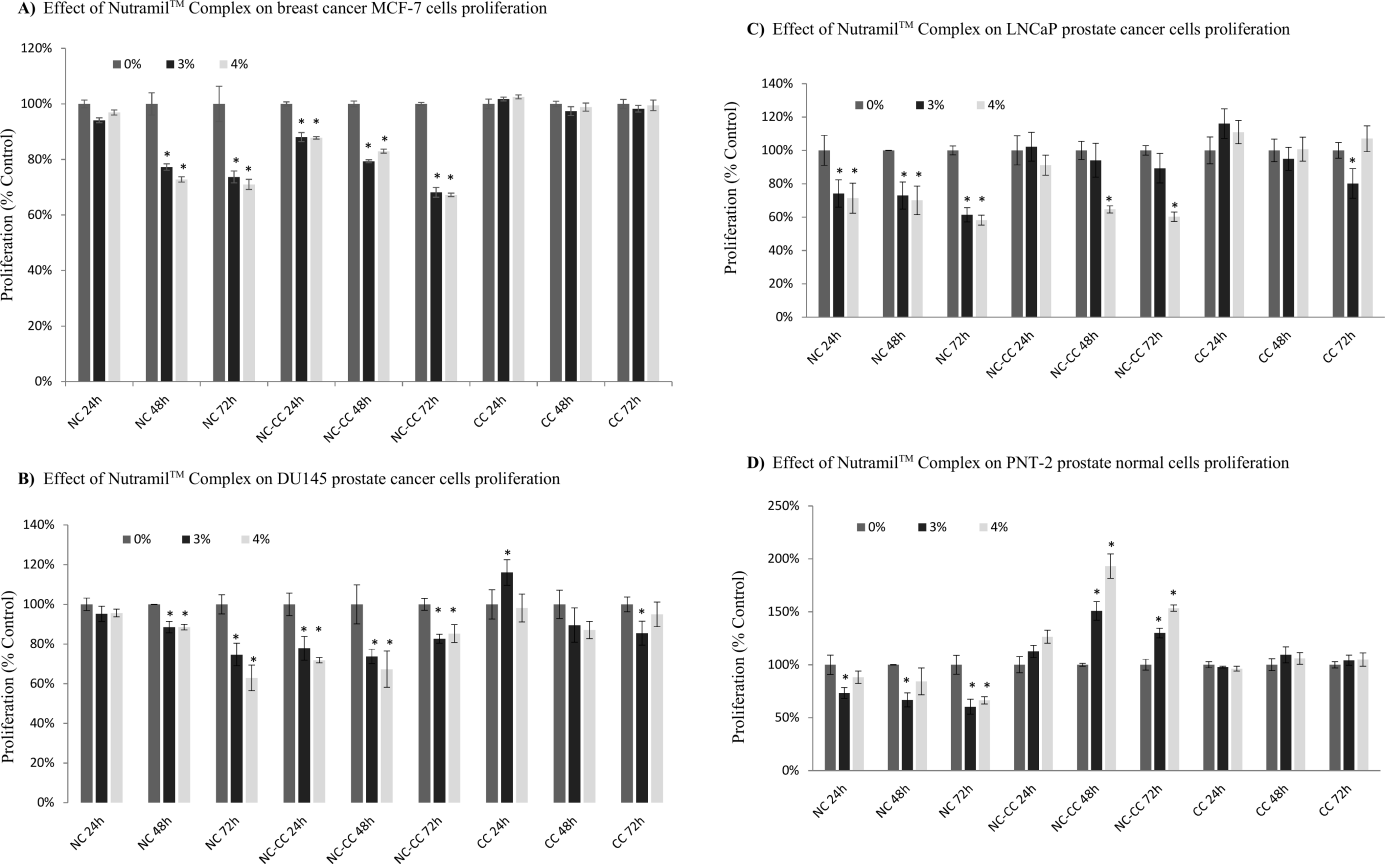
Effect of Nutramil^TM^ Complex on cells proliferation. MCF-7 breast cancer cells (A), DU145 prostate cancer cells (B), LNCaP prostate cancer cells (C) and PNT-2 normal prostate cells (D) were seeded on the 96-well plates (8x10^3^ cells per well). 24 h after seeding, growth medium was replaced with a medium containing Nutramil^TM^ Complex (NC) or Nutramil^TM^ Complex without calcium caseinate (NC-CC) or calcium caseinate (CC) at concentration 0, 3, 4% for 24–72 h. Cell proliferation was measured using Cell Proliferation ELISA, BrdU (Roche, Poland). Values are expressed as mean ± SD for n = 12, standardized to untreated control (UC) as 100%. Statistical significance was based on t-test *P≤0.01 vs. UC.

BrDU labeling results showed that Nutramil^TM^ Complex reduced the proliferation of studied cell lines in a dose–and time–dependent manner. For MCF-7 breast cancer cells, proliferation was reduced by approximately 30% at 48–72 h (P≤0.001, Fig 2A). Similar trend was observed for the hormone-independent DU145 prostate cancer cell line, where proliferation decreased by 40% at 72 h post-treatment (Fig 2B). Most prominent were results for LNCaP (androgen-dependent) prostate cancer cells that showed the highest level of susceptibility to NC in our study. Reduction in proliferation by 30% was observed already at 24–48 h and reached 40–45% at 72 h post-treatment (Fig 2C). Interestingly, PNT-2 normal prostate cells reacted similarly to cancer cells and showed approximately 30–40% reduction in proliferation after treatment with NC (Fig 2D).

Results for NC-CC showed a similar trend to the NC treatment (Fig 2A–2C). Almost 30–35% reduction in proliferation for MCF-7 and DU145 cell line (P≤0.001; Fig 2A and 2B) and about 40% reduction after 72 h post-treatment for LNCaP cell line (P≤0.001; Fig 2C). On the other hand, proliferation of PNT-2 normal cell line significantly increased with time of treatment (P≤0.001; Fig 2D).

Treatment with CC did not show statistically different results for proliferation of MCF-7 and PNT-2 cells vs UC (Fig 2A and 2D); however, it did not promote proliferation of prostate cancer cells (Fig 2B and 2C).

### mRNA expression of genes associated with cell cycle and apoptosis

To further evaluate the effect of Nutramil^TM^ Complex on studied cancer cell lines, we investigated its effect on the expression of genes associated with cell cycle and apoptosis. Our analysis showed that treatment with NC has an effect on mRNA levels of multiple genes. All results are presented in Table 2.

**Table 2:** mRNA expression of genes associated with cell cycle and apoptosis in cancer cells.

Gene Symbol	DU145				LNCaP				MCF-7			
	NC vs UC		NC-CC vs UC		NC vs UC		NC-CC vs UC		NC vs UC		NC-CC vs UC	
	FC value	Adjusted p-values	FC value	Adjusted p-values	FC value	Adjusted p-values	FC value	Adjusted p-values	FC value	Adjusted p-values	FC value	Adjusted p-values
*AKT1*	-1,36	0,03214	-1,04	0,00055	1,18	0,00081	-1,29	0,02324	1,44	0,00007	1,67	0,01273
*APAF1*	2,02	0,03147	-1,01	0,02270	2,54	0,00698	-2,28	0,00287	1,19	0,00024	-2,85	0,00000
*BAD*	2,29	0,01694	1,84	0,00439	2,38	0,00589	2,16	0,00880	2,46	0,00006	3,15	0,02347
*BAX*	3,67	0,01097	2,54	0,00087	2,88	0,00430	1,71	0,00779	1,62	0,00000	1,43	0,00035
*BID*	3,52	0,01590	2,04	0,00133	2,47	0,00474	1,35	0,32196	1,25	0,00005	1,21	0,00023
*BCL2*	-2,54	0,00431	-3,24	0,00000	-1,15	0,00474	-1,55	0,00312	-2,76	0,00160	-7,35	0,00018
*CASP3*	13,14	0,00003	6,07	0,00000	2,82	0,03960	-1,34	0,00876	Ns	-	Ns	-
*CASP8*	5,68	0,00018	2,69	0,00053	3,05	0,00767	1,54	0,89843	2,23	0,00293	-1,14	0,00049
*CDKN2A*	3,23	0,00020	2,29	0,00008	3,00	0,00023	1,96	0,02395	Ns	-	Ns	-
*CYCS*	4,82	0,00908	2,70	0,00007	1,81	0,00112	1,95	0,00007	-3,11	0,00006	-4,47	0,00001
*FADD*	4,72	0,00174	2,47	0,00096	2,80	0,00072	-1,71	0,00178	2,30	0,00003	1,92	0,01687
*FAS*	5,20	0,01975	1,28	0,30215	3,02	0,02340	1,70	0,00566	-2,67	0,00233	-4,87	0,00002
*HRAS*	-1,42	0,68056	-1,03	0,00173	-1,47	0,00041	-2,04	0,00554	1,28	0,00000	1,35	0,00097
*IGF1R*	-1,12	0,00309	-1,41	0,00114	-1,06	0,00027	-1,81	0,00106	2,09	0,00001	1,49	0,00044
*KRAS*	2,85	0,00005	1,43	0,00010	-1,08	0,01581	-2,11	0,00009	-1,15	0,00005	-3,45	0,00026
*MYC*	1,03	0,00907	1,03	0,00032	-1,21	0,00120	1,13	0,01434	2,83	0,00011	2,00	0,01215
*NRAS*	2,04	0,00007	1,27	0,00047	-1,24	0,01742	-1,87	0,00803	-1,38	0,00002	-4,53	0,00001
*RRAS*	-1,25	0,58008	-1,35	0,07026	-1,47	0,03462	-1,48	0,07072	1,89	0,00047	1,83	0,01436
*TP53*	1,20	0,27491	1,21	0,00218	2,70	0,00292	1,02	0,28757	2,07	0,00000	1,94	0,00606
*YWHAB*	-1,06	0,00153	1,03	0,00014	-1,31	0,00269	-1,03	0,52029	1,50	0,00001	1,04	0,00017
*YWHAE*	1,38	0,00004	1,28	0,00000	-1,37	0,00001	-1,82	0,00639	-1,39	0,00000	-3,66	0,00000
*YWHAG*	-1,07	0,02792	1,02	0,01573	-1,55	0,00759	-1,36	0,25445	2,51	0,00133	1,58	0,01705
*YWHAH*	-1,17	0,04692	-1,08	0,02815	-1,48	0,00188	-1,56	0,08087	1,13	0,00055	-1,31	0,00166
*YWHAQ*	1,15	0,00036	1,06	0,00049	-1,32	0,00222	-1,55	0,00328	-1,03	0,00000	-3,06	0,00000
*YWHAZ*	1,43	0,00043	1,13	0,00279	-1,50	0,00172	-2,14	0,00291	1,36	0,00014	-2,92	0,00001

*AKT1*, Serine/Threonine Kinase 1; *APAF1*, Apoptotic Peptidase Activating Factor 1; *BAD*, Bcl2-Associated Agonist Of Cell Death; *BAX*, BCL2 Associated X, Apoptosis Regulator; *BID*, BH3 Interacting Domain Death Agonist, *CASP3*, Caspase 3; *CASP8*, Caspase 8; *CDKN2A*, Cyclin Dependent Kinase Inhibitor 2A; *CYCS*, Cytochrome C; *FADD*, Fas- Associated Death Domain; *FAS*, Fas Cell Surface Death Receptor; *HRAS*, HRas Proto-Oncogene, GTPase; *IGF1R*, Insulin Like Growth Factor 1 Receptor; *KRAS*, KRAS Proto-Oncogene, GTPase; *MYC*, MYC Proto-Oncogene, BHLH Transcription Factor; *NRAS*, NRAS Proto-Oncogene, GTPase; *RRAS*, Related RAS Viral (R-Ras) Oncogene Homolog; *TP53*, Tumor Protein P53; *YWHAB*, Tyrosine 3-Monooxygenase/Tryptophan 5-Monooxygenase Activation Protein Beta; *YWHAE*, Tyrosine 3Monooxygenase/Tryptophan 5-Monooxygenase Activation Protein Epsilon; *YWHAG*, Tyrosine 3Monooxygenase/Tryptophan 5-Monooxygenase Activation Protein Gamma; *YWHAH*, Tyrosine 3Monooxygenase/Tryptophan 5-Monooxygenase Activation Protein Eta; *YWHAQ*, Tyrosine 3Monooxygenase/Tryptophan 5-Monooxygenase Activation Theta; *YWHAZ* Tyrosine 3Monooxygenase/Tryptophan 5-Monooxygenase Activation Zeta.

Ns, no signal; Statistical significance of treatment: P ≤0.05.

For DU145, results showed down-regulation of anti-apoptotic *AKT-1*, *BCL-2*, *HRAS*, *IGF1R* and some members of the *YWHA* family as well as up-regulation of many pro-apoptotic genes, including *APAF1*, *BAD*, *BAX*, *BID*, *CASP3*, *CASP8*, *CDKN2A*, *CYCS*, *FADD*, *FAS* and *TP53*.

For LNCaP cells, results showed down-regulation of anti-apoptotic *HRAS*, *BCL-2*, *IGF1R*, *KRAS*, *MYC*, *NRAS*, *RRAS* and all members of the YWHA family as well as up-regulation of *APAF1*, *BAD*, *BAX*, *BID*, *CASP3*, *CASP8*, *CDKN2A*, *CYCS*, *FADD*, *FAS* and *TP53*.

For MCF-7 breast cancer cells, data showed a significant decrease in the mRNA levels of *BCL-2*, *KRAS* and *NRAS* oncogenes and some of the *YWHA* family. Pro-apoptotic genes, including *APAF1*, *CASP8*, *BID*, *BAD*, *BAX*, and *TP53* were up-regulated.

Similar trends were observed for treatment with NC-CC for all examined cell lines.

### Expression of proteins involved in cellular stress and apoptosis signaling

**Fig 3:**
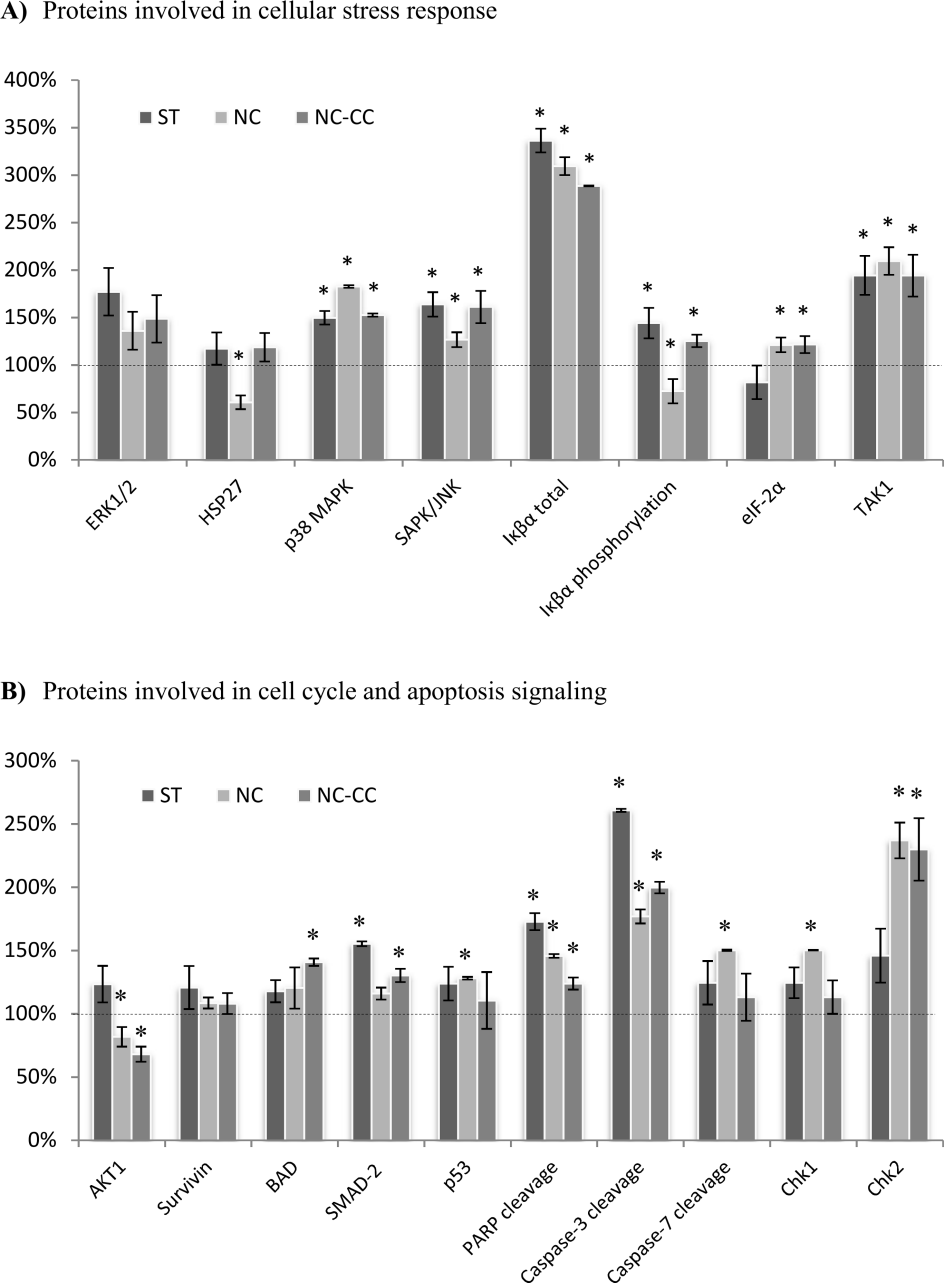
Expression of proteins involved in cellular stress and apoptosis signaling in MCF-7 breast cancer cells.

**Fig 4:**
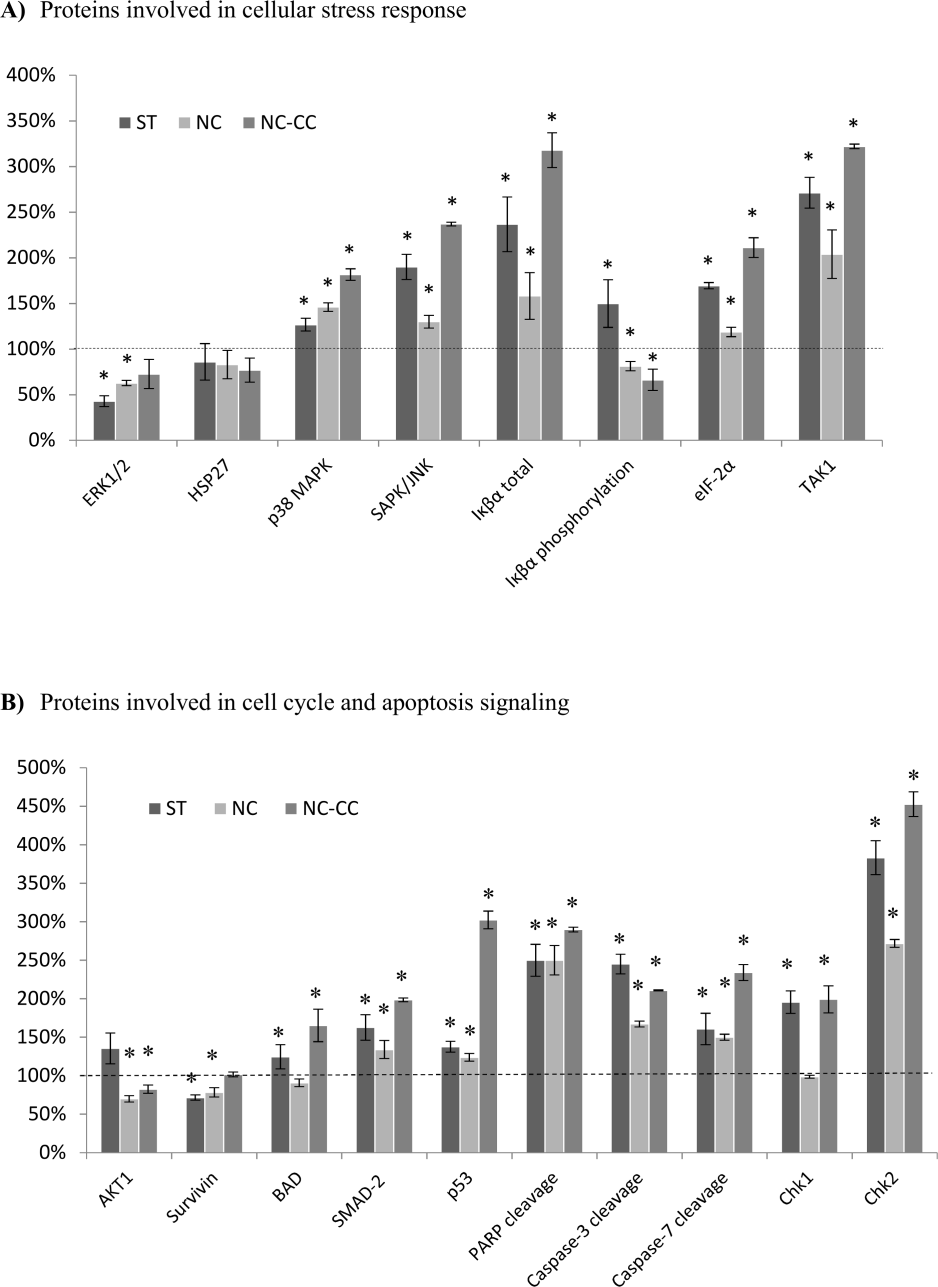
Expression of proteins involved in cellular stress and apoptosis signaling in DU145 prostate cancer cells. MCF-7 breast cancer (Fig 3) and DU145 prostate cancer (Fig 4) cells were treated for 48 h with NC, Nutramil^TM^ Complex, at 4% concentration; NC-CC, Nutramil^TM^ Complex without calcium caseinate, at 4% concentration or ST, staurosporine positive control, at 1.5 μM concentration. Cell extracts were prepared and analyzed using the PathScan® Stress and Apoptosis Signaling Antibody Array Kit (Chemiluminescent Readout; #12856, Cell Signaling Technology, MA, USA). Images were acquired by briefly exposing the slide to standard chemiluminescent film. Densitometry analysis was performed using ImageJ. Results are shown as a mean±SD normalized to the internal reference protein (α-Tubulin). Untreated negative control (NC) was set as 100% expression level. Statistical significance of NC was based on t-test *p≤0.05 vs. UC. (A)Proteins involved in cellular stress response. P44/42 MAPK (ERK1/2) phosphorylation (Thr202/Tyr204), HSP27 phosphorylation (Ser82), p38 MAPK phosphorylation (Thr180/Tyr182), SAPK/JNK phosphorylation (Thr183/Tyr185), IkB total, IkBα phosphorylation (Ser32/36), eIF-2α phosphorylation (Ser51), TAK1 phosphorylation (Ser412).(B)Proteins involved in cell cycle and apoptosis signaling. AKT phosphorylation (Ser473), Survivin total, BAD phosphorylation (Ser136), SMAD-2 phosphorylation (Ser465/467), p53 phosphorylation (Ser15), PARP cleavage (Asp214), Caspase-3 cleavage (Asp175), Caspase-7 cleavage (Asp198), Chk1 phosphorylation (Ser345), Chk2 phosphorylation (Thr68).

PathScan® Stress and Apoptosis Signaling Antibody Array analysis was performed for MCF-7 (Fig 3A and 3B) and DU145 (Fig 4A and 4B) to further investigate the effect of Nutramil^TM^ Complex on proteins involved in cellular stress, cell cycle and apoptosis signaling.

**Fig 5:**
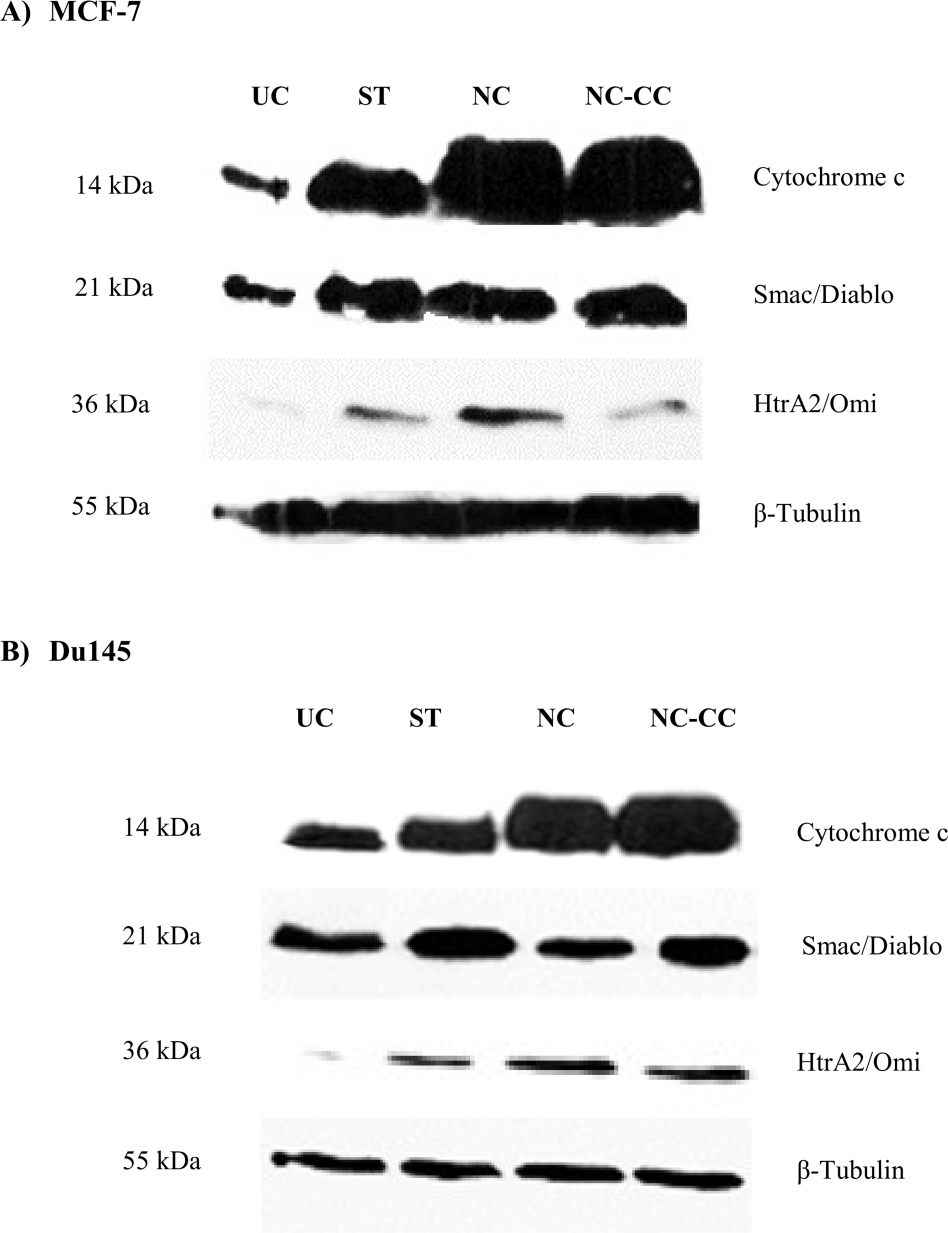
Expression of Cytochrome c, Smac/Diablo and HtrA2/Omi in cancer cells. MCF-7 breast cancer (A) and DU145 prostate cancer (B) cells were treated for 48 h with NC, Nutramil^**TM**^ Complex, at 4% concentration; NC-CC, Nutramil^**TM**^ Complex without calcium caseinate, at 4% concentration; or ST, staurosporine positive control, at 1.5 μM concentration. Cell extracts were prepared using Cell Lysis Buffer (Cell Signaling Technology, MA, USA) with the addition of Protease Inhibitor Cocktail (BioShop, Canada). Protein extracts were then separated on a polyacrylamide gel and transferred to a nitrocellulose filter (Bio-Rad, CA, USA) by wet-electroblotting. The immobilized proteins were incubated with Cytochrome c (#11940), Smac/Diablo (#2954), and HtrA2/Omi (#9745) primary antibody (Cell Signaling Technology, MA, USA). β-Tubulin (#2128, Cell Signaling Technology, MA, USA) was used as a reference protein. Detection was executed by chemiluminescence, using Clarity™ Western ECL Substrate (Bio-Rad, CA, USA).

In addition, levels of Cytochrome c, Smac/Diablo and HtrA2/Omi mitochondrial proteins were measured (Fig 5A and 5B).

For MCF-7 breast cancer cells, Nutramil^TM^ Complex reduced significantly the expression of pro-survival AKT1 down to 82% of UC (P≤0.05; Fig 3B). The same trend was found for the NC-CC treated cells (68% of UC, P≤0.05; Fig 3B). For pro-survival HSP27, results showed a significant decrease in protein expression only for NC treated cells (60% of UC; P≤0.05; Fig 3A). No changes were measured for Survivin (Fig 3B). Results for TAK1 showed a significant increase in the level of active protein form for both NC and NC-CC treated cells (209% and 194% of UC, respectively; P≤0.01; Fig 3A). Nutramil^TM^ Complex had a significant effect on the p38 MAPK level (183% of UC; P≤0.05; Fig 3A) as well as on SAPK/JNK MAP (127% of UC; P≤0.01; Fig 3A). Similar effects for both p38 MAPK and SAPK/JNK MAP were observed for NC-CC (152% of UC and 161% of UC respectively; P≤0.01; Fig 3A). Significant increase of total Iκβα was measured for both NC and NC-CC treated MCF-7 cells (309% of UC and 288% of UC; P≤0.01; Fig 3A). However, results for NC showed a significant reduction in levels of the phosphorylated form of Iκβα to 72% of NC (P≤0.05; Fig 3A). EIF-2α is the protein required to initiate translation processes. Our results showed a slight increase in phosphorylated protein form in cells treated with both NC and NC-CC (both 121% of UC; P≤0.05; Fig 3A).

Our results showed strong activation of pro-apoptotic proteins in MCF-7 cells treated by Nutramil^TM^ Complex, including p53 (128% of UC; P≤0.05; Fig 3B). Caspase-3 and Caspase-7 cleavage was significantly increased after NC treatment (177% and 150% of UC, respectively P≤0.05; Fig 3B). These results were also statistically significant for NC-CC, for which Caspase -3 protein expression levels were 200% of UC (P≤0.01; Fig 3B). In addition, results showed an increase in cleaved form of PARP after both NC and NC-C treatment (146% of UC and 124% of UC, P≤0.01, respectively; Fig 3B). Our results also showed an increase in protein levels of Cytochrome c, Smac/Diablo and HtrA2/Omi (Fig 5A), which promote caspases activity. The effect of NC-CC on the expression of apoptosis markers was similar to that of NC (Fig 5A), with the exception of HtrA2/Omi (Fig 5A). Chk1 and Chk2 kinases play an important role in DNA damage checkpoint control. Results for both showed a significant increase in protein expression levels after NC treatment (150% of UC and 237% of UC; P≤0.01; Fig 3B). Similar trend was observed for NC-CC treated cells (113% of UC and 230% of UC, respectively Fig 3B).

For DU145 cells, treatment with Nutramil^TM^ Complex reduced the level of pro-survival proteins such as ERK1/2 to 63% of UC (P≤0.05; Fig 4A), AKT1 to 70% of UC (P≤0.05; Fig 4B), HSP27 to 83% of UC (NS; Fig 4A), Survivin to 78% of UC (P≤0.01; Fig 4B), phospho-BAD to 90% of UC (NS; Fig 4B) and phospho-Iκβα to 81% of UC (P≤0.05; Fig 4A). Expression of TAK1 was increased to 204% of UC (P≤0.05; Fig 4A). On the other hand, the expression of pro-apoptotic proteins, p53 and SMAD-2, was increased (124% and 134% of UC, respectively; P≤0.05; Fig 4B). Caspase-3 and Caspase-7 cleavage was found increased to 167% and 150% of UC respectively (P≤0.01; Fig 4B). In addition, results also showed increased levels of cleaved PARP cleavage (250% of UC; P≤0.01; Fig 4B) as well as mitochondrial Cytochrome c and HtrA2/Omi (Fig 5B). Total Iκβα increased to 158% of UC (P≤0.05; Fig 4A), p38 MAPK to 146% of UC (P≤0.05; Fig 4A) and SAPK/JNK to 130% of UC (P≤0.05; Fig 3B). Significant increase in protein expression was found for Chk2 (272% of UC; P≤0.01; Fig 4A); however, NC had no measurable effect on Chk1 level (99% of UC; Fig 4B). EIF-2α protein expression was 119% of UC after NC treatment (P≤0.05; Fig 4A).

The effect of the Nutramil^TM^ formulation without calcium caseinate on pro-survival proteins was similar to that of NC (Fig 4A and 4B). Down-regulation in protein levels was measured for ERK1/2 (73% of UC; P≤0.05; Fig 4A), AKT1 (82% of UC; P≤0.05; Fig 4B), HSP27 (77% of UC; NS; Fig 4A), phospho-Iκβα (66% of UC; P≤0.05; Fig 4A) as well as up-regulation of TAK1 (322% of UC; P≤0.05; Fig 4A) and phospho-BAD (165% of UC; P≤0.05; Fig 4B). The effect of NC-CC on the expression of pro-apoptotic proteins was stronger than of NC (Fig 5B), again with the exception of HtrA2/Omi (Fig 5B). Expression of tumor suppressors p53 and SMAD-2 increased respectively to 302% and 198% of UC (P≤0.01; Fig 4B); levels of Caspase-3 cleavage increased to 211%, Caspase-7 cleavage to 234% and PARP cleavage to 290% of UC (P≤0.01; Fig 4B). Similarly, results showed an increase in expression of p38 MAPK (182% of UC; P≤0.05; Fig 4A), SAPK/JNK (237% of UC; P≤0.05; Fig 4A) as well as total Iκβα (318% of UC; (P≤0.01; Fig 4A). Finally, NC-CC treatment resulted in upregulation of Chk1 and Chk2 kinases (199% and 453% of UC, respectively; P≤0.01; Fig 4B) and eIF-2α (211% of UC; P≤0.01; Fig 4A). NC-CC treatment did not seem to have any effect on Survivin levels (101% of UC; Fig 4B).

## Discussion

Nutramil^TM^ Complex is a complete nutritional supplement, providing about 417 kcal/100 g from 60% carbohydrate, 25% fat and 15% protein. It was designed to contain all essential nutrients i.e. basic nutrients, minerals and vitamins in balanced proportions as required by dietary recommendations. The high-quality protein is delivered in the form of calcium caseinate, which is a milk protein. Carbohydrates, including sugars, are supplied in form of maize maltodextrin with varying absorption rates. The product is clinically free of lactose. As sources of fats include: rapeseed oil (80%) and medium-chain triglycerides (MCTs) (20%). It provides very important mono-unsaturated oleic acid, essential fatty acids, as well as long-chain triglycerides (LCTs). MCTs provide fast and lasting energy and are easily absorbed by the body. In addition, they also have a positive effect on calcium absorption.

**Fig 6:**
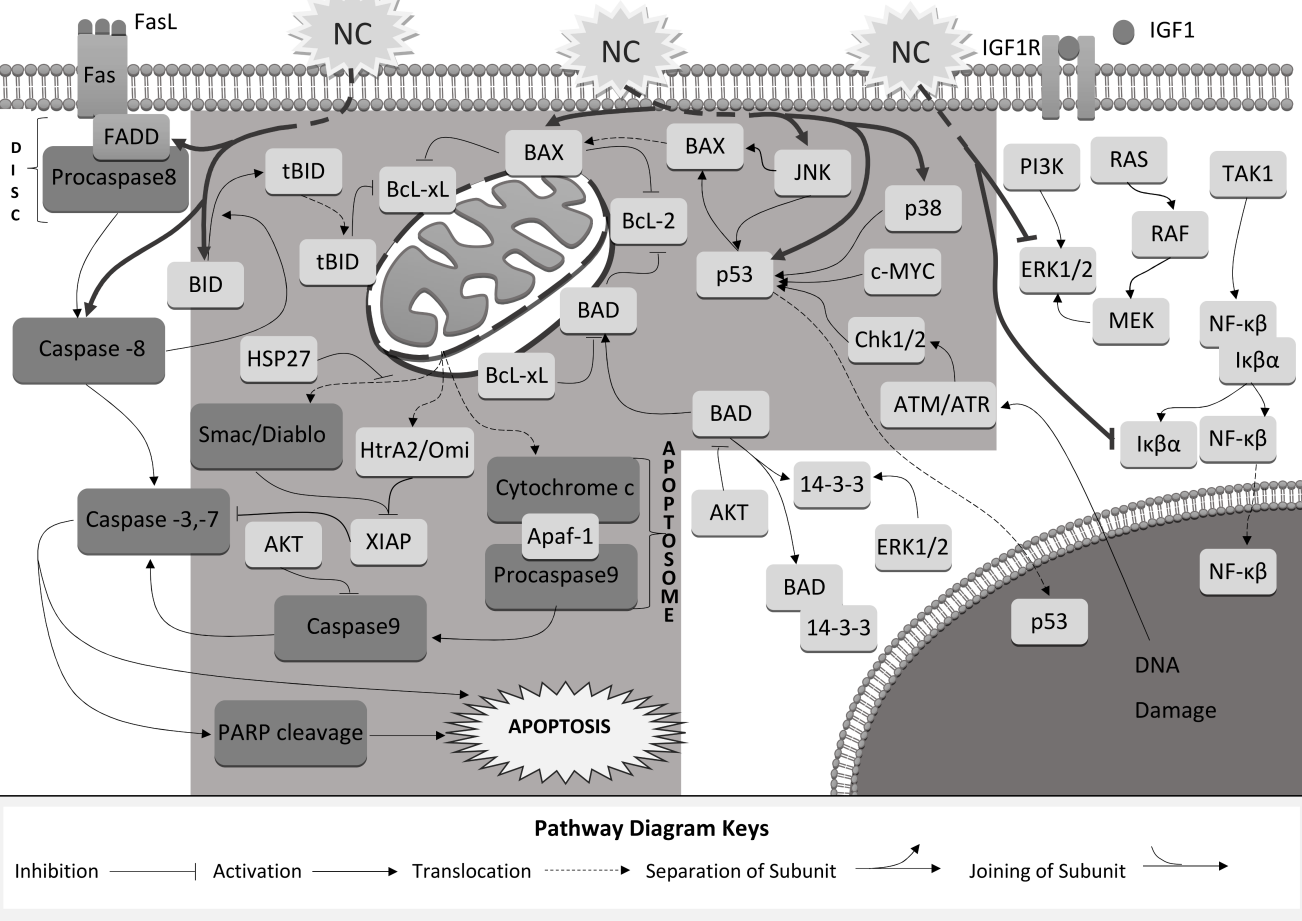
Mechanism of NC induced apoptosis in cancer cells.

In our previous pilot studies, we have shown that Nutramil^TM^ Complex decreases the viability of breast and prostate cancer cells [7, 8]. In current manuscript, we made an attempt to determine the potential molecular mechanism after treatment with NC (Figs 1A–1C and 2A–2C). To examine whether the apoptosis was the primary cause of cell death, we analysed the levels of genes associated with apoptotic events. Further, we verified the effect of NC on selected proteins involved in cellular stress signalling related to apoptosis induction (Fig 6).

Our results for NC treated cancer cells showed an increased expression of *TP53* in all examined cell lines. We also measured promoted expression of *CDKN2A* in prostate cancer lines (Table 2). The *TP53* gene is a key cancer suppressor gene. The p53 protein controls the transcription of many different genes in response to stress signals, thus regulating processes related to DNA repair, cell-cycle arrest or apoptosis. In turn, the *CDKN2A* codes for p16 and p14ARF proteins that act via two independent pathways: retinoblastoma RB- and p53- tumour suppressor pathways, respectively [10]. Chk1 and Chk2 play an important role in DNA damage checkpoint control (Fig 6). They also affect the post-translation modifications of p53 protein, leading to its accumulation [11]. Our results confirm over-expression of p53 protein after NC treatment (Figs 3B and 4B) as well as an increase in Chk1 and Chk2 protein expression (Figs 3B and 4B). As a transcription factor, p53 is responsible, among others, for the regulation of mitochondrial-induced pro-apoptotic proteins of BCL-2 family [12,13]. BCL-2 is a family of proteins regulating the apoptosis processes by controlling the mitochondrial permeability [14]. Our results showed that NC increased gene expression of pro-apoptotic *BAX*, *BID*, *BAD* members, and decreased expression of anti-apoptotic *BCL-2* (Table 2). In addition, we determined up-regulation of *APAF1* and *CYCS* genes (Table 2), which encodes a cytoplasmic protein that initiates apoptosis (Fig 5). Apaf-1 protein is a caspase-activating molecule that is released from the mitochondria during apoptosis induction. Activation of p53 results in oligomerization of pro-apoptotic members (BAX, BID, PUMA, NOXA) at the mitochondrial outer membrane and translocation of Cytochrome c and Apaf-1 into cytosol followed by activation of Caspases [15]. The Caspase-3 and Caspase-7 are the downstream effector caspases that cleave specific cellular targets. PARP is one of the earliest nuclear enzymes to be targeted by Caspase-3 during apoptosis [16]. In our study, we confirmed the activation of pro-apoptotic markers (Cytochrom c, Smac/Diablo, HtrA2/Omi) as well as Caspase-3 and 7 under NC (Figs 3B, 4B, 5A and 5B). We also measured an increased level of cleaved form of PARP (Figs 3B and 4B).

In our work, we also verified the impact of NC on selected proteins involved in cellular stress response and their links to apoptosis induction (Fig 6). Our results showed that NC treatment decreased expression of ERK1/2 (Fig 4A) and AKT1 (Fig 4B) proteins in prostate cancer cells and AKT1 and HSP27 cells in MCF-7 cells (Fig 3A and 3B). AKT kinase is a major signaling node that transmit signals related to many essential pro-survival cellular functions. The main consequence of its activation is increased proliferation and tumor transformation and inhibition of apoptosis [17,18]. In addition, AKT phosphorylated the pro-apoptotic BAD protein at Ser136 and inhibits its ability to induce apoptosis [19,20]. HSP27 is a mediator of cellular stress that confers resistance to adverse environmental conditions. It was associated with the inhibition of Cytochrome c release from mitochondria and with an increase in AKT expression [21].

Our results showed a significantly elevated level of phosphorylated p38 MAPK and SAPK/JNK MAP kinases in both MCF-7 and DU145 cell lines after the treatment with NC (Figs 3A and 4A). Activation of p38 MAPK and SAPK/JNK MAP kinases occurs via dual phosphorylation mechanism in response to cellular stressors and leads to regulation of the inflammatory response, cell-cycle arrest and apoptosis induction. It has been suggested that SAPK/JNK can contribute to the both, the c-Jun / AP-1 external apoptosis pathway as well as to the mitochondria-dependent apoptosis pathway via p53 and pro-apoptotic BCL-2 family activation. In addition, the suggestion was made that these mechanisms can act independently or co-operate with one another to induce cell death [22]. Our results give support to this hypothesis as they showed an increased expression of *FADD* and *CASP 8* genes in both breast and prostate cancer cell lines and for the prostate cell lines also of *FAS* gene, associated with the external apoptosis pathway (Table 2).

Our results showed also an increase in *MYC* mRNA expression in both MCF-7 and DU145 lines, while its down-regulation in LNCaP (Table 2). *MYC* expression is closely associated with the progression of cell-cycle in normal tissues and its overexpression has been found in variety of cancer types. Interestingly, increasing number of research shows that *MYC* can be also involved in the control of apoptosis. McMahon [15] in his review points out that for normal cells when growth factors are limiting, cells with high *MYC* levels may activate p53 protein, leading to the induction of apoptotic events. However, in the absence of p53 activation and / or pre-dominance of pro-life factors such as *BCL-2*, high *MYC* expression may be insufficient to trigger apoptosis. Our results, demonstrated overexpression of *MYC*, p53 accumulation as well as lowered levels of *BCL-2* expression, supporting this hypothesis.

In our study, we also investigated the effect of NC on the activity of Iκβα. Iκβα protein regulates the activity of NF-κβ transcription factor by forming a protein complex that prevents translocation of NF-κβ to the nucleus. Presence of phosphorylated form of Iκβα indicates the degradation of the NF-κβ / Iκβα complex and translocation of NF-κβ into the nucleus, where as a transcription factor it can activate many pro- or anti-apoptotic genes [23]. Treatment with Nutramil^TM^ Complex significantly increased the level of un-phosphorylated Iκβα, capable of binding to NF-κβ (Figs 3A and 4A). Interestingly, our results showed elevated level of TAK1 protein, which may promote phosphorylation of Iκβα protein [24] while measuring lower levels of phosphorylated Iκβα after NC treatment (Figs 3A and 4A).

Many studies are available on individual components of Nutramil^TM^ Complex that show their potential anti-cancer properties. For MCT fats, they have been shown to delay tumour growth in a mouse xenograft model [25] and to exacerbate the therapeutic effect of studied anti-cancer substances in TCC cells in dogs [26]. Similarly, rape seeds have been shown to exhibit very strong antioxidant activity and have the potential to inhibit the proliferation of tumour cells [27, 28]. Studies in MCF-7 and T47D breast lines showed their inhibitory effect on cancer cell growth leading to induction of apoptosis via increased expression of Caspase-3 and p53 [29]. In the *in vivo* studies, canola oil diet significantly reduced the incidence as well as multiplicity of colorectal tumours in rats [30].

Nutramil^TM^ Complex contains all vitamins and minerals, including macronutrients and micronutrients essential for proper functioning of the human body (S1 Table). Some of them, including vitamin D, C, K, E, B complex vitamins and minerals, i.e. selenium, zinc and iodine, have proven anti-tumour activity [31–33]. Deeb et al. [34] have demonstrated that the active form of vitamin D3 (1α, 25 (OH) 2D3) exhibits anti-tumour properties by regulating the expression of Bcl-2 family proteins and by activating the Caspases. Vitamin D3 in Nutramil^TM^ is in the form of cholecalciferol, thus the pro-apoptotic effect of 1α,25(OH)2D3 may not be directly translated into the observed properties of the formulation; however, some studies suggest that cholecalciferol may be metabolized in some tumour cells, especially in breast cancer cells [35]. Studies *in vitro* on lymphoma cells using ascorbic acid (form of vitamin C present in Nutramil^TM^ Complex) showed a decrease in the viability of cancer cells without affecting normal cells [36]. In turn, vitamin K affects the levels of tyrosine kinases, phosphatases and activation of transcription factors Myc and Fos, followed by the regulation of expression of genes involved in cell cycle regulation and apoptosis induction [37].

For B complex vitamins, deficiencies in B_6_ and B_12_ can cause DNA damage and lead to cancer [38, 39]. Riboflavin reduces the risk of cancer by acting as a cofactor in folate metabolism [40] and by enhancing the anti-cancer activity of vitamin C [41]. The effect of thiamine supplementation is not yet fully understood. On the one hand, it has been shown that thiamine can enhance cancer cell proliferation and increase therapeutic resistance [42, 43]; on the other hand, a high dose of this vitamin has been shown to have an inhibitory effect on tumour cell growth [42, 44]. Vitamin E includes a vast group of compounds belonging to the tocopherols and tocotrienols family, which have been shown to have anti-tumour properties [45]. It has been shown that some vitamin E components such as RRR-alpha-tocopheryl succinate and tocopherol ether may have pro-apoptotic effect on cancer cells in a dose that does not affect healthy cells [46]. However, there are no reports linking the DL-α-tocopheryl acetate, which is an active vitamin E form in the Nutramil^TM^ Complex, with reduced survival of cancer cells. Another component of Nutramil^TM^ Complex is the sodium selenite, which anti-tumor properties have been demonstrated in animals [47] and in cell lines including fibrosarcoma, lung carcinoma and acute promyelocytic leukemia [48–51]. One of *in vivo* studies involved nude mice bearing human colorectal carcinoma SW480 cell line xenografts, which were injected peritoneally for 21 days with sodium selenite. Results showed significant tumour suppression. In addition, authors showed reduced expression of Bcl-xL proteins and increased expression of pro-apoptotic proteins, including Bax, Bad and Bim as well as Caspase-9 [47]. In studies using U2OS cells, induction of apoptosis has been shown to be associated with activation of Caspase-3, up-regulation of *TP53* and *PTEN* suppressors and down-regulation of *BCl-2* expression. In promyelocytic leukemia NB4 cells, sodium selenite induced reactive oxygen species generation that was associated with early events that triggers endoplasmic reticulum stress mitochondrial apoptotic pathways [51]. Finally, for A549 human lung carcinoma cells, sodium selenite has been shown to modulate both extrinsic and intrinsic apoptotic pathways. Apoptosis induction was mediated by reactive oxygen species (ROS) and was dependent on the activation of Caspases [49].

Zinc whose anti-neoplastic properties have been described for breast cancer cells and prostate cancer [52] is also present in the formulation. In both cell lines, apoptosis was associated with the mitochondrial pathway, including events such as activation of pro-apoptotic BAX, Cytochrome c translocation and Caspase activation. In breast cancer cells (MCF-7) induction of apoptosis was accompanied by accumulation of p53 protein [53], whereas in PC-3 prostate cancer cells, apoptosis was independent of p53, but also via the mitochondrial pathway [52, 54]. Another mineral ingredient of the Nutramil^TM^ formulation with potential anti-neoplastic significance is magnesium. This element participates in many metabolic processes and redox reactions [55]. Magnesium present in drinking water was associated with reduction of the risk of liver cancer [56], while high-dose magnesium supplementation was shown to reduce the risk of colorectal cancer [57]. Epidemiological studies confirm the relationship between magnesium deficiency and colorectal cancer in overweight people [58]. It is also worth mentioning the role of calcium ions in regulation of proliferation and induction of apoptosis. Regulation of calcium ion concentration is dependent on activation of ion channels, including K^+^ and Cl^-^ channels, that regulate the membrane potential and increase the probability of Ca^2+^ channel opening. Also, the activity of individual ions affects their concentration in the cytoplasm [59]. The correlation has been shown between deregulation of these ions and apoptosis. Increasing intracellular chloride and sodium ion concentrations results in increased ROS, release of Cytochrome c into the cytoplasm and cell apoptosis [60]. Finally, among the listed mineral components of the preparation, iodine also has been shown to have a pro-apoptotic activity on breast cancer (MCF-7) [61].

In conclusion, our study, as one of very few, show the effect of complete food medical supplement on cancer cells. It is well known that cells integrate multiple signals from a variety of sources before following either pro- or anti-apoptotic pathway. Taking into account the significant reduction if cancer cell growth (Figs 1A–1C and 2A–2C) with simultaneous activation of many pro-apoptotic proteins, tumour suppressors and regulators of cell-cycle (Figs 3A, 3B, 4A. 4B, 5A and 4B and Table 2), it can be concluded that Nutramil^TM^ Complex exhibits anti-tumor properties. Moreover, our data suggest that it activates apoptotic events via mitochondrial-induced pathway (Fig 6). This is most likely related to the composition of the preparation: the optimum proportion, bioavailability and cellular metabolism of the individual components. It appears that Nutramil^TM^ Complex as a Food for Special Medical Purpose can support the treatment of oncological patients, not only due to their complete nutritional value, but also for the cytotoxic effects on tumour cells. We admit that our results are based on the *in vitro* model which requires further verification *in vivo*; However, according to the 3R principles (Replacement, Reduction and Refinement), the *in vitro* studies are a valuable and multi-faceted source of information and may explain the adequacy of further *in vivo* research.

## Supporting information

S1 Table Composition of Nutramil ^TM^ Complex as food for special medical purpose. (DOCX)
