# Supplementary material for: The effect of “NutramilTM Complex,” food for special medical purpose, on breast and prostate carcinoma cells
Source: PLoS One. 2018 Feb 14;13(2):e0192860. doi: 10.1371/journal.pone.0192860 (PMC5812662; doi:10.1371/journal.pone.0192860)
Supplement: S1 Table — (DOCX) [file pone.0192860.s001.docx]

**Tabel 1S.** Composition of Nutramil^TM^ Complex as Food for Special Medical Purpose

| **Nutritional value per 100g of product** | | | | | |
| --- | --- | --- | --- | --- | --- |
| **Energy Value** | | | | 1743kJ/ 417kcal | |
| **Nutrients** | | | | | |
| Total Carbohydrates  - Sugars  - Lactose | | | | 62,5 g  7,2 g  <0,08 g | |
| Protein | | | | 15,6 g | |
| Total Fat  - Saturated Fatty Acids  - Medium*-*Chain Triglycerides MCT | | | | 11,7 g  3,5 g  2,4 g | |
| **Mineral components** | | **Vitamins** | | | |
| Potassium  Calcium  Sodium  Chlorides  Phosphorus  Magnesium  Zinc  Iron  Manganese  Copper  Iodine  Selenium  Molybdenum  Chromium | 485 mg  253 mg  236 mg  222 mg  194 mg  63 mg  2,8 mg  2,1 mg  0,56 mg  278 µg  42 µg  15 µg  14 µg  11 µg | Fat-Soluble | Vitamin E  Vitamin A  Vitamin K  Vitamin D | | 3,3 mg  222 µg  21 µg  1,4 µg |
|  |  | Water-Soluble | Vitamin C  Niacin  Pantothenic acid  Vitamin B_2_  Vitamin B_6_  Vitamin B_1_  Folic acid  Biotin  Vitamin B_12_ | | 22 mg  4,4 mg  1,7 mg  0,4 mg  0,4 mg  0,3 mg  56 µg  13,9 µg  0,7 µg |
